# Tuberculosis in the colombian-venezuelan border: geospatial distribution

**DOI:** 10.17843/rpmesp.2022.393.11249

**Published:** 2022-09-30

**Authors:** Silvia Liliana Ruíz-Roa, Sandra Milena Martínez-Rojas, Iván Andrés Felipe Serna-Galeano

**Affiliations:** 1 Public Health Research Group-GISP, Faculty of Health Sciences, Universidad Francisco de Paula Santander, Cúcuta, Colombia. Universidad Francisco de Paula Santander Public Health Research Group-GISP Faculty of Health Sciences Universidad Francisco de Paula Santander Cúcuta Colombia; 2 Nursing Care Research Group - GICE, Faculty of Health Sciences, Universidad Francisco de Paula Santander, Cúcuta, Colombia. Universidad Francisco de Paula Santander Nursing Care Research Group - GICE Faculty of Health Sciences Universidad Francisco de Paula Santander Cúcuta Colombia; 3 Laboratory of Automation, Systems, Embedded and Robotics-LASER, Engineering School, Universidad Distrital Francisco José Caldas, Bogotá, Colombia. Universidad Distrital Francisco José de Caldas Laboratory of Automation, Systems, Embedded and Robotics-LASER Engineering School Universidad Distrital Francisco José Caldas Bogotá Colombia

**Keywords:** Geographic Information Systems, Spatial Analysis, Cluster Analysis, Geographic mapping, Tuberculosis, Morbidity

## Abstract

The geospatial distribution of pulmonary and extrapulmonary tuberculosis (TB) morbidity in the municipality of Cúcuta in 2019 and 2020 was described by the Kulldorff method using the geographic location and reporting date of incident TB cases. The unit of analysis was the event reported to the National Epidemiological Surveillance System (SIVIGILA). A total of 607 cases were identified in 392 neighborhoods distributed in ten communes. Most cases of pulmonary TB were reported in the northern commune, with the El Salado neighborhood being the most affected repeatedly. Incident cases of extrapulmonary TB did not show patterns of repetition in the distribution between spatial and temporal units. Strategies to mitigate and control the spread of pulmonary infection should prioritize the western region.

## INTRODUCTION

According to the World Health Organization (WHO), tuberculosis (TB) is a disease that can be found in all countries and age groups, being one of the first ten causes of mortality worldwide with 1.5 million deaths in 2020 ^1^. In Colombia, case reporting has increased, with an incidence rate of 26.9 cases per 100,000 population for 2018 ^2^. The border state of Norte de Santander has an incidence rate of 38.8 cases per 100,000 inhabitants ^3^, with indicators higher than the national ones for the same year.

The geospatial analysis of events of public health interest allows the identification of disease dynamics, the most susceptible geographic areas, and the local, social, and environmental characteristics that generate greater risks for the population group of acquiring or presenting a disease in a given period ^4^. These are fundamental aspects in diseases such as TB, where the establishment of these dynamics can favor control strategies and mitigation of its spread ^5^.

Several studies have used spatial analysis to identify the economic, social and environmental factors that influence the morbimortality of the population in the territories ^6^. Among these, the Kulldorff test, which uses a consecutive circular scan in specific geographic regions to identify clusters, calculating the probability ratio according to the number of cases observed within that perimeter in each circle ^7^, complementing the processes of monitoring, evolution, establishment of transmission patterns and control of the spread of infectious diseases of public health interest such as TB ^8^. This allows formulating and directing effective strategies to meet the needs of the territory and contribute to the mitigation of the disease and improve the quality of life of the population.

Despite the multiple benefits of geographic analysis of events of public health interest, there is no evidence of these in the Colombian-Venezuelan border, disregarding this resource for the formulation of health action plans in the municipality of Cúcuta, which could favor their implementation by the local government. Therefore, this study aimed to describe the geospatial distribution of TB cases in the municipality of Cúcuta (Colombia) in 2019-2020.

**KEY MESSAGES**

Motivation for the study: In Cúcuta, a Colombian-Venezuelan border city, tuberculosis represents a serious public health problem that mainly affects the poorest communities with limited access to the healthcare system.

Main findings: We established the spatial and temporal distribution of pulmonary tuberculosis, which showed a greater repetition of cases in the northern region of the municipality for two consecutive years; however, extrapulmonary tuberculosis did not show marked differences between spatial and temporal units in the same period.

Implications: It is necessary to strengthen the implementation of measures of health promotion, mitigation, and control of the spread of infection with a multidisciplinary and multisectoral approach in the most affected regions.

## THE STUDY

### Design, scope, and unit of analysis

Descriptive, retrospective, cross-sectional study that described the geospatial distribution of TB cases presented in the municipality of Cúcuta in the years 2019 and 2020, by generating clusters using the Kulldorff test.

The geographic area of Cúcuta is composed of 392 formally recognized and grouped neighborhoods, comprising ten urban communes, which are named according to their geographic location ^9^.

The unit of analysis was the epidemiological records reported to the National Epidemiological Surveillance System (SIVIGILA) following confirmed diagnosis of TB in the healthcare institutions of the municipality of Cúcuta. We excluded TB cases whose address or neighborhood of residence was not registered.

SIVIGILA is the body of the Colombian Ministry of Health responsible for the permanent observation and analysis of events of public health interest, consolidating data on the cases reported by each territorial unit ^10^. For this study, the Departmental Health Institute of Norte de Santander (IDS) granted the research team access to the event data by an institutional permit.

### Study variables

The analysis was made up of variables grouped into four categories as specified below:

*Temporal presentation*

We considered the epidemiological week and the year of notification of each incident TB case to SIVIGILA.

*Sociodemographic profile*

Age, sex (male and female), housing stratification (type 1, 2, 3, 4, 5 or 6), nationality and type of affiliation to the health system (contributory or subsidized).

*Geographic profile*

Locality of the household stratified by neighborhood, commune, and exact address of residence.

*Clinical profile of the TB case*

We considered the condition of the disease (sensitive or resistant to drug therapy); the anatomical location of the infection (pulmonary or extrapulmonary); previous anti-TB treatment (yes and no) and comorbidities and/or coexistence of other diseases (diabetes, HIV, among others).

### Statistical analysis

Categorical variables were presented with absolute and relative frequencies which are described in tables.

We used the Kulldorff method ^7^ to construct the spatial distribution of the data used to generate the clusters, by ranking the number of events observed in a given period, to form intensity diagrams of the neighborhoods and communities most affected by the presence of TB repeatedly over time. In order to generate the distribution maps of incident and prevalent TB cases (considering those reported to SIVIGILA in previous quarters in the same epidemiological year), we used geographic information software. In this case, we used the Qgis version 3.4 free software to carry out the spatial analysis of the .shp, .kml, or GeoJson type information, depending on the source of the data, generating choropleth maps and identifying the neighborhoods and communes where the event was most frequent.

The repetition of cases in the clusters defined by the different neighborhoods was established following a gradual colorimetric pattern, where the lightest color represents the absence of TB cases; and the highest color intensity identified the neighborhoods most affected with the event in a repetitive manner in a certain period of time.

### Ethical aspects

The information was processed preserving confidentiality ^11^ and was used exclusively for achieving the objectives of the study. This research was approved by the Ethics Committee of the Faculty of Health Sciences (CEIV-15-2021-ENFERMERIA). In addition, to avoid stigmatization of the cases due to their location, the geospatial analysis was carried out taking as a reference the location of the events, grouping them by neighborhoods, without schematizing them by blocks or exact place of residence.

## FINDINGS

A total of 607 cases of tuberculosis were reported. Age was stratified by groups, with most events concentrated in the 25-34 years age range (25.7%). Most TB cases occurred in men (68.5%), of Colombian nationality (86.6%), who accessed the subsidized health system (52.5%). Table 1 shows the demographic profile of TB cases.

Clinical variables showed that 539 (88.8%) cases had pulmonary and 68 (11.2%) extrapulmonary infection, most with drug-sensitive infections (98.8%), no co-infection with human immunodeficiency virus (HIV) (93.4%) and no hospitalization during treatment (53.7%). Table 2 compares the clinical profiles of TB cases reported in 2019 and 2020.

**Table 1:** Sociodemographic variables of incident TB cases. Cúcuta, 2019-2020.

Variable	2019		2020		
	n	%	n	%
Total	325	100	282	100
Age (years)				
0-14	3	0.9	4	1.4
15-24	50	15.4	51	18,1
25-34	74	22.8	82	29.2
35-44	49	15.1	51	18.1
45-54	43	13.2	21	7.4
55-64	39	12.0	29	10.2
≥ 65	67	20.6	44	15.6
Sex				
Female	107	32.9	84	29.7
Male	218	67.1	198	70.3
Nationality				
Venezuelan	18	5.5	30	10.6
Colombian	277	85.2	249	88.2
Not identified	30	9.3	3	1.2
Health insurance				
Special	5	1.5	0	0.0
Exception	56	17.2	48	17.1
Contributive	56	17.2	49	17.4
Subsided	171	52.6	148	52.5
Not determined	5	1.5	1	0.3
Not insured	32	10.0	36	12.7

Source: Public health surveillance system, SIVIGILA.

**Table 2:** Distribution of incident TB cases according to their clinical characteristics. Cúcuta, 2019-2020.

Variable	2019		2020	
	n	%	n	%
Clinical form				
Pulmonary	288	88.7	251	89.1
Extrapulmonary	37	11.3	31	10.9
Pharmacological condition				
Sensitive	321	98.8	279	98.9
Resistant	4	1.2	3	1.1
HIV co-infection				
Yes	22	6.8	18	6.4
No	303	93.2	264	93.6
Hospitalized/inpatient				
Yes	143	44.0	138	48.9
No	182	56.0	144	51.1

Source: Public health surveillance system, SIVIGILA.

We found that the neighborhoods most affected by the event were those located in the North commune (23.3%), followed by the West commune (14.9%), Southwest (12.6%) and Northwest (11.8%), concentrating 62.1% and 63.8% of the cases reported in each year, respectively.

**Figure 1:**
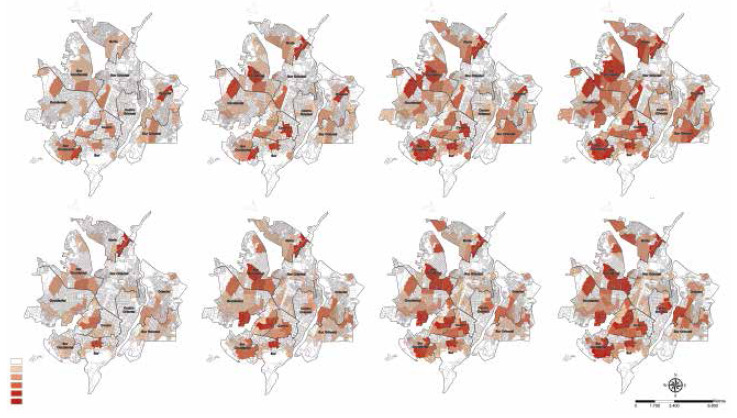
Geospatial distribution of spatiotemporal clusters of cumulative cases of pulmonary tuberculosis in the city of Cúcuta, Colombia, 2019-2020.

**Figure 2:**
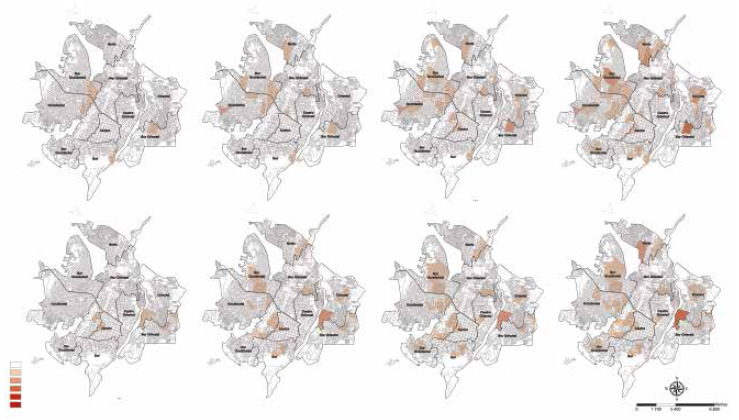
Geospatial distribution of spatiotemporal clusters of cumulative cases of extrapulmonary tuberculosis in the city of Cúcuta, Colombia, 2019-2020.

TB cases were geocoded and represented temporally by quarter for the years 2019 and 2020. Figures 1 and 2 represent the choropleth maps of cumulative pulmonary and extrapulmonary TB cases reported in Cúcuta, Colombia, for each year respectively. By using the Kulldorff method, we found a non-random distribution of the occurrence of pulmonary TB, evidencing that most of the incident and prevalent cases as of the second quarter of 2019 were repetitively concentrated in the El Salado neighborhood of the North commune with 15.0%. Similarly, the temporal evolution of the occurrence for pulmonary TB in 2020 showed that cases were concentrated in the El Salado neighborhood of the North commune from the beginning to the end of the epidemiological year with 16.3% (46) of the events reported to SIVIGILA (Figure 1).

The analysis of the temporal and geographic evolution of extrapulmonary TB cases in 2019 and 2020 did not show a defined distribution pattern maintained over time as occurred with pulmonary TB.

## DISCUSSION

Our findings showed that most cases of pulmonary TB occurred repeatedly in the El Salado neighborhood of the North commune during the two studied years, which was not the case for extrapulmonary TB, where the cases did not show a spatial and temporal distribution pattern.

Demographically, the cases were more frequent among the young adult male population, those with Colombian nationality and those who accessed the subsidized health care system. The most frequent form of TB was pulmonary, with drug-sensitive infections, without co-infection with HIV, and did not require hospitalization during treatment. These demographic and clinical characterization data are similar to those previously described in a state capital of southern Brazil, where TB cases between 2011-2013 were predominantly male, with pulmonary infection and, to a lesser extent, HIV coinfection ^12^.

Several studies have described that non-spatial variables such as age, sex, household characteristics and low socioeconomic status can influence infection rates and the outcome of TB treatment ^13^ ^-^ ^15^. However, it is important to note that despite being the Colombian territory most affected by the migratory flow of Venezuelans, which has impacted the poverty and overcrowding rates in the region ^16^, the rates of multidrug-resistant TB are low ^17^, favoring the control and mitigation of the spread of the disease in this territory.

This study identified communities and neighborhoods with the highest frequency of TB cases in the municipality of Cúcuta, the Colombian-Venezuelan border capital. Cases of pulmonary TB were mostly located in the northern area of the municipality of Cúcuta, from the second quarter of 2019 to December 2020. We found higher and repeated frequencies of the event in the El Salado neighborhood, being this neighborhood of low socioeconomic stratum and high poverty conditions, which establishes the importance of prioritizing this territory within local policy actions.

Based on our findings and the existence of global strategies for the prevention and control of TB ^18^ adopted by the national government with projected interventions until the year 2025 ^19^, it is necessary to prioritize the implementation of preventive measures and direct efforts for their use, with greater emphasis on the most affected geographic areas. In order to reach this goal, it is necessary to formulate specific and precise public health information, intervention, evaluation, and surveillance systems in accordance with the needs of the population, as well as the maintenance of geographic monitoring mechanisms for follow-up.

The results obtained with the current analysis are pioneering in the region; however, there are some limitations related to the use of secondary data registered in SIVIGILA. The main limitation is the data management system, in which the information is subjected to processes of double digitization and manual correction, among others; a process that is completed at the end of the first quarter of the following year, which is why the data for the year 2021 were not included in this analysis. Based on the above, there is an inability to guarantee the correct filling out of the notification forms and/or data base entry, which can produce problems secondary to incorrect or incomplete data, underreporting and loss of information ^20^ ^-^ ^22^; however, the source of these data is the government entity responsible at the regional level, adding credibility to the findings. The results presented here can serve as a tool to establish the regional distribution patterns of TB incidence in the near future, in order to compare them with future studies, to evaluate the results of interventions for TB control, and to direct and promote strategies against the spread of the infection in the Colombian-Venezuelan border region.

In conclusion, the geographic distribution of pulmonary TB cases during 2019 and 2020 was predominantly concentrated in the western region of the municipality of Cúcuta, with repeating patterns in the northern commune during the two consecutive years. These findings suggest the importance of using geospatial analysis in addressing public health problems in the region, with the provision of economic, human, and logistical resources targeted to the most affected populations to reduce or eradicate the impact of TB in the municipality.
