# Supplementary material for: Tuberculosis in the colombian-venezuelan border: geospatial distribution
Source: Rev Peru Med Exp Salud Publica. 2022 Sep 30;39(3):362–7. doi: 10.17843/rpmesp.2022.393.11249 (PMC11397580; doi:10.17843/rpmesp.2022.393.11249)
Supplement: Translation

# Translation: Tuberculosis en la frontera colombo-venezolana: distribución geoespacial

**Authors:** Silvia Liliana Ruíz-Roa, Sandra Milena Martínez-Rojas, Iván Andrés Felipe Serna-Galeano

**Affiliations:** 1 Grupo de Investigación de Salud Pública-GISP, Facultad Ciencias de la Salud, Universidad Francisco de Paula Santander, Cúcuta, Colombia.; 2 Grupo de Investigación Cuidado de Enfermería-GICE, Facultad Ciencias de la Salud, Universidad Francisco de Paula Santander, Cúcuta, Colombia.; 3 Laboratorio de automatización, sistemas, embebidos y robótica-LASER, Facultad de Ingenierías, Universidad Distrital Francisco José Caldas, Bogotá, Colombia.

**Keywords:** Sistemas de información geográfica, Análisis espacial, Análisis por conglomerados, Mapeo geográfico, Tuberculosis, Morbilidad

## Abstract

La distribución geoespacial de la morbilidad por tuberculosis (TB) pulmonar y extrapulmonar en el municipio de Cúcuta en los años 2019 y 2020 se describió utilizando el método de Kulldorff usando la ubicación geográfica y la fecha de reporte de los casos incidentes de TB. La unidad de análisis fue el evento reportado al Sistema Nacional de Vigilancia Epidemiológica (SIVIGILA). Se identificaron 392 barrios distribuidos en diez comunas, donde se ubicaron 607 casos. En la distribución de la TB pulmonar, la comuna norte fue la que más casos reportó, siendo el barrio El Salado el más afectado de manera repetitiva. Los casos incidentes de TB extrapulmonar no mostraron patrones de repetición en la distribución entre las unidades espaciales y temporales. Se debe priorizar la región occidental dentro de las estrategias de mitigación y control de la propagación de la infección pulmonar del territorio.

## INTRODUCCIÓN

Según la Organización Mundial de la Salud (OMS), la tuberculosis (TB) es una enfermedad presente en todos los países y grupos de edad, siendo una de las primeras diez causas de mortalidad a nivel mundial con 1,5 millones defunciones en el 2020 ^1^. En Colombia, la notificación de casos se incrementó, con una tasa de incidencia de 26,9 casos por cada 100 000 habitantes para el 2018^ (^ ^2^. El estado fronterizo de Norte de Santander presenta una tasa de incidencia de 38,8 casos por 100 000 habitantes ^3^, con indicadores superiores a los nacionales para el mismo año.

El análisis geoespacial de los eventos de interés en salud pública es una estrategia que permite identificar la dinámica de las enfermedades, las áreas geográficas más susceptibles, las características locales, sociales y ambientales que generan mayores riesgos para el grupo poblacional de adquirir o presentar una enfermedad en un determinado periodo ^4^; aspectos fundamentales en enfermedades como la TB, donde el establecimiento de esta dinámica puede favorecer las estrategias de control y mitigación de su propagación ^5^. 

Diversos estudios han utilizado el análisis espacial para identificar los factores económicos, sociales y ambientales que influencian la morbimortalidad de la población en los territorios ^6^; entre estos, la prueba de Kulldorff, que realiza un escaneo circular consecutivo en regiones geográficas específicas para identificar conglomerados, calculando en cada círculo la razón de probabilidad según el número de casos observados dentro de ese perímetro ^7^, complementando los procesos de acompañamiento, evolución, establecimiento de patrones de transmisión y control de propagación de enfermedades infectocontagiosas de interés de salud pública como la TB ^8^. Esto permite formular y direccionar estrategias eficaces que satisfagan necesidades propias del territorio que contribuyan a la mitigación de la enfermedad y a mejorar la calidad de vida de las poblaciones.

A pesar de los múltiples beneficios del análisis geográfico de los eventos de interés de salud pública, no existe evidencia de estos en la frontera colombo-venezolana, desconsiderando este recurso para la formulación de planes de acción en salud del municipio de Cúcuta, que puedan favorecer su implementación por el gobierno local. Por lo tanto, el objetivo de este trabajo fue describir la distribución geoespacial de los casos por TB en el municipio de Cúcuta (Colombia) en 2019-2020.

**MENSAJE CLAVE**

Motivación para realizar el estudio: Cúcuta es la ciudad fronteriza colombo-venezolana donde la tuberculosis representa un serio problema de salud pública que afecta mayoritariamente a las comunidades más pobres y con limitado acceso al sistema de salud.

Principales hallazgos: se estableció la distribución espacial y temporal de la tuberculosis pulmonar con una mayor repetición de casos en la región norte del municipio por dos años consecutivos; sin embargo, la tuberculosis extrapulmonar no mostró diferencias marcadas entre unidades espaciales y temporales en el mismo periodo.

Implicancias: es necesario intensificar la implementación de medidas de promoción de salud, mitigación y control de la propagación de la infección con abordaje multidisciplinar y multisectorial en las regiones más afectadas.

## ESTUDIOS

### Diseño, ámbito de estudio y unidad de análisis

Estudio descriptivo, retrospectivo, transversal que permitió describir la distribución geoespacial de los casos de TB presentados en el municipio de Cúcuta en los años 2019 y 2020, a través de la generación de conglomerados empleando la prueba de Kulldorff.

El área geográfica de Cúcuta está compuesta por 392 barrios formalmente reconocidos y agrupados, integrando diez comunas urbanas, las cuales reciben su nombre de acuerdo con su localización geográfica ^9^. 

Se empleó como unidad de análisis las fichas epidemiológicas reportadas al Sistema Nacional de Vigilancia Epidemiológica (SIVIGILA) levantadas tras el diagnóstico confirmado de TB en las instituciones prestadoras de servicios de salud del municipio de Cúcuta. Fueron excluidos los casos de TB cuya dirección o barrio de residencia no estuviese registrado. 

El SIVIGILA es el órgano del Ministerio de Salud colombiano responsable por la observación y análisis permanente de los eventos de interés de salud pública, consolidando los datos de los casos notificados por cada unidad territorial ^10^. Para el presente estudio, el Instituto Departamental de Salud de Norte de Santander (IDS), concedió, a través de un permiso institucional, acceso a los datos del evento al equipo investigador. 

### Variables de estudio

El análisis realizado estuvo integrado por variables agrupadas en cuatro categorías como se especifica a continuación:

*Presentación temporal*

Fue considerada la semana epidemiológica y el año de notificación de cada caso incidente de TB ante el SIVIGILA.

*Perfil sociodemográfico*

Edad, sexo (masculino y femenino), estratificación de la vivienda (tipo 1, 2, 3, 4, 5 o 6), nacionalidad y tipo de filiación al sistema de salud (contributivo o subsidiado).

*Perfil geográfico*

Localidad de la vivienda estratificada en barrio, comuna y dirección exacta de residencia.

*Perfil clínico del caso de TB*

Se consideró la condición de la enfermedad (sensible o resistente a la farmacoterapia); la localización anatómica de la infección (pulmonar o extrapulmonar); la presencia de antecedente de tratamiento antituberculoso (sí y no) y comorbilidades y/o coexistencia de otras enfermedades (diabetes, VIH, entre otras).

### Análisis estadístico

Los datos de las variables categóricas se presentaron con frecuencias absolutas y relativas descritas en tablas. 

La distribución espacial de los datos para generar los conglomerados se realizó a través del método de Kulldorff ^7^, jerarquizando el número de eventos observados en determinado periodo, para formar diagramas de intensidad de los barrios y comunas más afectadas por la presencia de TB de manera repetida en el tiempo. Para generar los mapas de distribución de los casos incidentes y prevalentes (considerando los reportados al SIVIGILA en los trimestres anteriores en el mismo año epidemiológico) de TB, se utilizó un *software* especializado en sistemas de información geográfica, en este caso se utilizó el *software* libre Qgis versión 3.4 con el cual fue posible realizar el análisis espacial de la información de tipo .shp. .kml, o tipo GeoJson, según la fuente de estos, se generaron mapas coropléticos y se identificaron los barrios y comunas donde fue más frecuente el evento.

La repetición de los casos en los conglomerados definidos por los diferentes barrios fue establecida siguiendo un patrón colorimétrico gradual, donde el color más claro representa la ausencia de casos de TB; y la mayor intensidad de color identificó a los barrios más afectados con el evento de manera repetitiva en determinado periodo de tiempo.

### Aspectos éticos

La información se procesó preservando la confidencialidad ^11^ y para el logro exclusivo de los objetivos del estudio. La investigación fue avalada por el Comité de Ética de la Facultad Ciencias de la Salud (CEIV-15-2021-ENFERMERIA). Adicionalmente, para evitar estigmatización de los casos por su localización, fue realizado el análisis geoespacial teniendo como referencia la ubicación de los eventos, agrupándolos por barrios, sin esquematizarlos por manzanas o sitio exacto de residencia. 

## HALLAZGOS

Fueron notificados 607 casos de tuberculosis. La edad fue estratificada por grupos, concentrando la mayoría de los eventos en el rango de 25-34 años (25,7%). La mayoría de casos de TB se presentó en hombres (68,5%), de nacionalidad colombiana (86,6%), atendidos por el régimen subsidiado de salud (52,5%). La Tabla 1 evidencia el perfil demográfico de los casos de TB.

**Tabla 1:** Variables sociodemográficas de casos incidentes por TB. Cúcuta, 2019-2020.

Variable	2019		2020	
	n	%	n	%
Total	325	100	282	100
Edad (años)				
0-14	3	0,9	4	1,4
15-24	50	15,4	51	18,1
25-34	74	22,8	82	29,2
35-44	49	15,1	51	18,1
45-54	43	13,2	21	7,4
55-64	39	12,0	29	10,2
≥ 65	67	20,6	44	15,6
Sexo				
Femenino	107	32,9	84	29,7
Masculino	218	67,1	198	70,3
Nacionalidad				
Venezolana	18	5,5	30	10,6
Colombiana	277	85,2	249	88,2
No identificados	30	9,3	3	1,2
Aseguramiento en salud				
Especial	5	1,5	0	0,0
Excepción	56	17,2	48	17,1
Contributivo	56	17,2	49	17,4
Subsidiado	171	52,6	148	52,5
Indeterminado	5	1,5	1	0,3
No asegurado	32	10,0	36	12,7

Fuente: Sistema de vigilancia en salud pública, SIVIGILA.

Las variables clínicas mostraron que 539 (88,8%) casos presentaron infección pulmonar y 68 (11,2%) extrapulmonar, la mayoría, con infecciones sensibles al tratamiento farmacológico (98,8%), sin coinfección con el virus de la inmunodeficiencia humana (VIH) (93,4%) y sin internación hospitalaria durante el tratamiento (53,7%). La Tabla 2 compara los perfiles clínicos de los casos de TB notificados en los años 2019 y 2020.

**Tabla 2:** Distribución de casos incidentes por TB según sus características clínicas. Cúcuta, 2019-2020.

Variable	2019		2020	
	n	%	n	%
Forma clínica				
Pulmonar	288	88,7	251	89,1
Extrapulmonar	37	11,3	31	10,9
Condición farmacológica				
Sensible	321	98,8	279	98,9
Resistente	4	1,2	3	1,1
Coinfección con VIH				
Sí	22	6,8	18	6,4
No	303	93,2	264	93,6
Hospitalizado/internado				
Sí	143	44,0	138	48,9
No	182	56,0	144	51,1

Fuente: Sistema de vigilancia en salud pública, SIVIGILA.

Se observó que los barrios más afectados por el evento fueron los localizados en la comuna Norte (23,3%), seguidos de la comuna Occidental (14,9%), Suroccidental (12,6%) y Noroccidental (11,8%), concentrando 62,1 y 63,8% de los casos notificados en cada año, respectivamente.

Los casos de TB fueron geocodificados y representados temporalmente por trimestre de los años 2019 y 2020. Las Figuras 1 y 2 representan los mapas coropléticos de los casos acumulados de TB pulmonar y extrapulmonar reportados en Cúcuta, Colombia, para cada año respectivamente. A través de la aplicación del método de Kulldorff, se observó una distribución no aleatoria de la ocurrencia de TB pulmonar, evidenciando que la mayoría de los casos incidentes y prevalentes a partir del segundo trimestre de 2019 estuvieron concentrados de manera repetitiva en el barrio El Salado de la comuna Norte con el 15,0%. De igual manera, la evolución temporal de la ocurrencia por TB pulmonar en el año 2020 mostró que los casos estuvieron concentrados en el barrio El Salado de la comuna Norte desde el inicio hasta el final del año epidemiológico con 16,3% (46) de los eventos notificados al SIVIGILA (Figura 1).

**Figura 1:** Distribucion geoespacial de los conglomerados espaciotemporales de casos acumulados de tuberculosis pulmonar en la ciudad de Cucuta, Colombia, 2019-2020

**Figura 2:** Distribucion geoespacial de los conglomerados espaciotemporales de casos acumulados de tuberculosis extrapulmonar en la ciudad de Cucuta, Colombia, 2019-2020.

Respecto a los casos de TB extrapulmonar en 2019 y 2020, el análisis de su evolución temporal y geográfica no mostró un patrón de distribución definido y mantenido en el tiempo como ocurrió con la TB pulmonar.

## DISCUSIÓN

Los hallazgos mostraron que la mayoría de los casos de TB pulmonar se presentaron de manera repetitiva en el barrio El Salado de la comuna Norte durante los dos años estudiados, aspecto que no se reprodujo con la TB extrapulmonar, donde los casos no mostraron un patrón de distribución espacial y temporal.

Demográficamente, los casos fueron más frecuentes en la población adulta joven, del sexo masculino, nacionalidad colombiana y atendidos por el régimen de atención en salud subsidiado. La forma de TB más frecuente fue la pulmonar, con infecciones sensibles a la medicación, sin coinfección con el VIH, y no requirieron internación hospitalaria durante el tratamiento. Estos datos de caracterización demográfica y clínica son semejantes con los descritos previamente en una capital de estado del Sur de Brasil, donde los casos de TB presentados entre 2011-2013, fueron predominantemente del sexo masculino, con infección pulmonar y en menor proporción con coinfección con VIH ^12^.

Diversos estudios han descrito que variables no espaciales como edad, sexo, características del domicilio y bajo nivel socioeconómico, pueden influenciar las tasas de infección y el resultado del tratamiento de la TB ^13^ ^-^ ^15^. Sin embargo, se destaca que a pesar de ser el territorio colombiano más afectado con el flujo migracional de venezolanos que ha impactado los índices de pobreza y hacinamiento de la región ^16^, las tasas de TB resistentes a múltiples fármacos es baja ^17^, favoreciendo el control y mitigación de la propagación de la enfermedad en este territorio.

El presente estudio identificó comunas y barrios con mayor frecuencia de casos de TB en el municipio de Cúcuta, capital fronteriza colombo-venezolana. Los casos de TB pulmonar se ubicaron mayormente en el área norte del municipio de Cúcuta, consolidando desde el segundo trimestre de 2019 hasta diciembre de 2020, mayores y repetidas frecuencias del evento en el barrio El Salado, siendo este barrio de estrato socioeconómico bajo y altas condiciones de pobreza, lo que establece la importancia de priorizar este territorio dentro de las acciones de políticas locales.

Considerando los hallazgos del presente estudio y la existencia de estrategias globales para la prevención y control de la TB ^18^, adoptadas por el gobierno nacional, con proyección de actividades hasta el año 2025 ^19^, es necesario priorizar la puesta en marcha de medidas preventivas y dirigir esfuerzos para su utilización, haciendo mayor énfasis en las áreas geográficas más afectadas. Para lograr esta propuesta se requiere la formulación de sistemas de información, intervención, evaluación y vigilancia en salud pública, específicos, precisos y acordes con las necesidades del territorio, así como el mantenimiento de mecanismos de monitoreo geográfico para su acompañamiento.

Los resultados obtenidos con el actual análisis son pioneros en la región; sin embargo, existen algunas limitaciones relacionadas con el uso de los datos secundarios registrados en el SIVIGILA, considerando como principal limitante el sistema de gestión de datos, en el cual la información obtenida es sometida a procesos de doble digitación y corrección manual, entre otros; proceso que se culmina al concluir el primer trimestre del año posterior, razón por la cual no fueron incluidos los datos del año 2021 en este análisis. Partiendo de lo anterior, existe la incapacidad de garantizar el correcto diligenciamiento de las fichas de notificación y/o digitación de las bases de datos que pueden generar problemas secundarios a datos incorrectos, incompletos, subregistro y pérdida de información ^20^ ^-^ ^22^; no obstante, la fuente de estos es la entidad gubernamental responsable a nivel regional, agregando credibilidad a los hallazgos. Los resultados aquí presentados pueden servir como herramienta que establezca los patrones de distribución regional de la incidencia de TB en un periodo próximo, que permitan compararlos con estudios futuros, así evaluar los resultados de intervenciones para el control de la TB, además, direccionar y promover estrategias contra la propagación de la infección en la región de frontera colombo-venezolana.

En conclusión, la distribución geográfica de los casos de TB pulmonar durante los años 2019 y 2020 se concentró predominantemente en la región occidental del municipio de Cúcuta, con patrones de repetición en la comuna norte durante los dos años consecutivos. Estos hallazgos sugieren la importancia de emplear el análisis geoespacial en el abordaje de los problemas de salud pública de la región, con la provisión de recursos económicos, humanos y logísticos dirigidos a las poblaciones más afectadas para reducir o erradicar el impacto de la tuberculosis en el municipio.
